# Danggui-Shaoyao-San Improves Gut Microbia Dysbiosis and Hepatic Lipid Homeostasis in Fructose-Fed Rats

**DOI:** 10.3389/fphar.2021.671708

**Published:** 2021-07-13

**Authors:** Jing Yin, Jiaxi Lu, Peng Lei, Mingshuai He, Shengjie Huang, Jialin Lv, Yan Zhu, Zhidong Liu, Miaomiao Jiang

**Affiliations:** ^1^ State Key Laboratory of Component-Based Chinese Medicine, Tianjin University of Traditional Chinese Medicine, Tianjin, China; ^2^ Department of Pharmacy, Institute of Traditional Chinese Medicine, Tianjin University of Traditional Chinese Medicine, Tianjin, China

**Keywords:** Danggui-Shaoyao-San, metabolic syndrome, gut microbiadysbiosis, anti-inflammation, hepatic lipid homeostasis

## Abstract

Metabolic syndrome (MetS) is a pathological state of many abnormal metabolic sections. These abnormalities are closely related to diabetes, heart pathologies and other vascular diseases. Danggui-Shaoyao-San (DSS) is a traditional Chinese medicine formula that has been used as a therapy for Alzheimer’s disease. DSS has rarely been reported in the application of MetS and its mechanism of how it improves gut microbia dysbiosis and hepatic lipid homeostasis. In this study, three extracts of DSS were obtained using water, 50% methanol in water and methanol as extracting solvents. Their chemical substances were analyzed by ultra-performance liquid chromatography coupled with quadrupole time-of-flight mass (UPLC-Q/TOF-MS). Pharmacodynamic effect of the extracts were evaluated by comparison of biochemical factors, 16S rRNA sequencing test for gut microbiota analysis, as well as metabonomic and transcriptomic assessments on liver tissues from fructose-fed rats. This study aimed at investigating DSS’s mechanism of regulating blood lipid, anti-inflammation and reducing blood glucose. The results showed that the 50% methanol extract (HME) was more effective. It was worth noting that hydroxysteroid 17β-dehydrogenase 13 (HSD17β13) as a critical element of increasing blood lipid biomarker-triglyceride (TG), was decreased markedly by DSS. The influence from upgraded hydroxysteroid 17β-dehydrogenase 7 (HSD17β7) may be stronger than that from downgraded *Lactobacillus* in the aspect of regulating back blood lipid biomarker-total cholesterol (TC). The differential down-regulation of tumornecrosis factor alpha (TNF-α) and the significant up-regulation of *Akkermansia* showed the effective effect of anti-inflammation by DSS. The declining glycine and alanine induced the lowering glucose and lactate. It demonstrated that DSS slowed down the reaction of gluconeogenesis to reduce the blood glucose. The results demonstrated that DSS improved pathological symptoms of MetS and some special biochemical factors in three aspects by better regulating intestinal floras and improving hepatic gene expressions and metabolites.

## Introduction

Metabolic syndrome (MetS) is a pathological state of many abnormal metabolic sections (Boehm and Claudi-boehm, 2005) such as overweight, dyslipidemia, hypertension, hyperuricemia and insulin resistance (IR). These abnormalities are closely related to the emergency with diabetes, especially with type 2 diabetes, heart pathologies and other vascular diseases.

Intestinal microorganisms refer to bacteria existing in the gut of the host body. They are involved in the pathogenesis of obesity or metabolic diseases. Intestinal flora is an important correlation between the gut and liver. Normally, only a small amount of bacteria and endotoxin enter into the liver through the portal vein, and later most of them are cleared by hepatic macrophages. Imbalance of intestinal flora results in the destruction of the intestinal barrier and the increase of bacteria. When bacterial metabolites exceed the scavenging capacity of the liver, through pattern recognition receptor, it can activate inflammatory reaction and accelerate hepatic disease progression (Wieland et al., 2015).

Danggui-Shaoyao-San (DSS) has been recorded by Zhongjing Zhang in *Jin Gui Yao Lue* (Synopsis of Prescriptions of the Golden Chamber) of the Eastern Han dynasty. It is used for bolstering the blood cycle and alleviating stasis due to blood stasis and deficiency of spleen Qi (Song, 2009). It consists of six herbals: smoke-dried root of *Angelica sinensis* (Oliv.) Diels (Danggui, Angelicae Sinensis Radix), sun-dried root of *Paeonia lactiflora* Pall. (Baishao, Paeoniae Radix Alba), oven-dried rhizome of *Ligusticum chuanxiong* Hort. (Chuanxiong, Chuanxiong Rhizoma), dried sclerotia of *Poria cocos* (Schw.) Wolf (Fuling, Poria), oven or sun dried rhizome of *Atractylodes macrocephala* Koidz. (Baizhu, Atractylodis macrocephalae rhizoma), and dried stem tuber of *Alisma plantago-aquatica* Linn. (Zexie, Alismatis Rhizoma). DSS is also known as Tokishakuyakusan in Japanese, which is one of Kampo formulas traditionally used for patients with irregular menstruation, fatigue, and anaemia (Kampo.ca Japanese Traditional Medicine & Therapeutics, 2014). In the 1980s, DSS was used in the treatment of dementia firstly reported by Japanese scientists (Mizushima and Ikeshita, 1989). Several clinical studies have reported that DSS provides reductions in symptoms of Alzheimer’s disease (AD) and vascular dementia (VD) making it as a promising anti-dementia drug candidate (Matsuoka et al., 2012; Fu et al., 2015; Kim and Cho, 2020). Moreover, the syndrome of spleen deficiency and fluid retention has been demonstrated as the important pathogenesis for the incidence and development of AD (Yu et al., 2014). Comorbidities of dementia include obesity, diabetes, hypertension, and cardiovascular diseases. The risk factors for these comorbidities are collectively referred to as MetS (Pugazhenthi, 2017). IR and visceral fat during MetS have been suggested to be important links between MetS and cognitive dysfunction (Ryu et al., 2019; Ivanova et al., 2020). Pharmacological intervention in MetS represents a risk reduction for mild cognitive impairment (MCI) and dementia including AD and VD later in life. Although there are numerous reviews about the application of DSS, this study is the first report of DSS on MetS. Herbal powders of DSS are inconvenient to take orally making the studies of the oral extracts more prevail. Numerous identified compounds have been reported from extracts of DSS (Chen et al., 2009; Niu and Wang, 2014). Monoterpenoid glycosides, phenolic acids, phthalides and lactones from DSS are active in protecting the cardiovascular system, including regulating blood lipid, preventing atherosclerosis and improving hemodynamics (Dong et al., 2014). Total glucosides of paeony including albiflorin and paeoniflorin from Baishao have significantly analgesic, anti-inflammatory and anticoagulant effects (Zhou et al., 2012; Shen et al., 2020). In this study, fructose-induced MetS rats were employed to evaluate the action of DSS extracts. We found that 50% methanol extract (HME) showed greater advantages in adjusting the biomarker parameters, the structure of intestinal flora. Subsequently, we studied the improvement of HME in hepatic metabolites and transcriptomic changes and found the internal connections. All the results revealed that HME could alleviate the MetS symptoms through a variety of interactions of hepatic metabolites, genes and intestinal microorganisms.

## Materials and Methods

### Materials

Danggui (Tongrentang, Beijing, China), Baishao (Tongrentang, Beijing, China), Chuanxiong (Tongrentang, Beijing, China), Fuling (Tongrentang, Beijing, China), Baizhu (Tongrentang, Beijing, China) and Zexie (Tongrentang, Beijing, China). All the traditional Chinese medicine decoction pieces were authenticated by Dr. Honghua Wu from Tianjin University of Traditional Chinese Medicine.

Adenosine (Meilun, Dalian, China), gallic acid (Meilun, Dalian, China), ferulic acid (Meilun, Dalian, China), albiflorin (Meilun, Dalian, China), paeoniflorin (Meilun, Dalian, China), atractylenolide I (Meilun, Dalian, China), alisol B-23-acetate (Meilun, Dalian, China), Z-ligustilide (Meilun, Dalian, China), Pioglitazone (Yuanye, Shanghai, China) (HPLC > 98%), D-fructose (Meilun, Dalian, China).

### Preparation for Fructose Solution and Test Drugs

Seven hundred grams of d-fructose was added into 3950 ml of water and the mixed solution was stirred until fructose was dissolved to yield 15% fructose water (w/w).

Danggui, Baishao, Chuanxiong, Fuling, Baizhu, and Zexie were finely grounded and mixed with a dose proportion of 450 g:1500 g:750 g:600 g:600 g:750 g. DSS was divided into several parts and every part was refluxed for 2 h by water, 50% methanol in water (v/v) or methanol, respectively. The extracting solutions were concentrated and freeze-dried to yield three extracts, water extract (WE), 50% methanol extract (HME) and methanol extract (TME). The yields of these dried extracts were all about 20%.

### Liquid Chromatography–Mass Spectrometry Analysis for Test Drugs

The qualitative analysis was carried out on Waters Xevo G2-S UPLC-Q-TOF/MS (Waters Milford, United States). The separation was carried out on a Waters ACQUITY UPLC BEH C18 (100 × 2.1mm, 1.7 μm) at 30°C. The mobile phase was 0.1% formic acid water (A) and methanol (B). The gradient program of mobile phase was as follows: 0–3 min, 5–14% B; 3–16 min, 14–56% B; 16–23 min, 56–80% B; 23–25 min, 80–95% B; 25–30 min, 95–5% B. The injection volume was 2 μl. The quasi-molecular ions including [M-H]^−^, [M-H+HCOOH]^−^, [M-H-CO_2_]^−^, [M-H-H_2_O]^−^, [M+H-H_2_O]^+^, [M+H]^+^ and [M+Na]^+^ were selected as precursor ions and subjected to target-MS/MS analyses. The acquisition parameters of Q/TOF were as follows: drying gas (N_2_) flow rate, 50 L/h; drying gas temperature, 400°C; the collision energy in high channels (MS^E^ pattern) was set at 20–60V; the mass range from m/z 50–100 Da Lock spray™ flow rate was 5 μl/min, and data acquisition was based on the Centroid Mode.

### Animals and Sample Collection

A total of 60 male Wistar rats (120–150 g per rat) were purchased from National Institutes for Food and Drug Control, License number: SCXK (Beijing) 2016-0006. The animal ethics approval number was TJAB-TJU20180041. Rats were maintained in SPF grade animal house in a 12-h dark-light cycle and had free access to food and water. Care and husbandry followed standard guidelines. After one week, rats were randomly and equally divided into six groups, including control group (fed with purified water; i.g. homologous saline per day), model group (fed with high-fructose drink; i.g. homologous saline per day), positive group (fed with high-fructose drink; i.g. 4 mg/kg dosage of pioglitazone), WE group (fed with high-fructose drink; i.g. 1.8 g/kg dosage of water extract), HME group (fed with high-fructose drink; i.g. 1.8 g/kg dosage of 50% methanol extract), TME (fed with high-fructose drink; i.g. 1.8 g/kg dosage of methanol extract). Treatments were continued for 8 weeks.

The body weight and the level of fasted blood glucose were measured every week. At the end of treatment, animals were kept in an empty cage without bedding to gather fresh stool samples into tubes. The rats were subjected to 12 h of fasting before they were sacrificed. Anaesthesia with 5% chloral hydrate was administered, blood was collected from the abdominal aorta and centrifuged to yield serum samples. The liver and intestine were precisely dissected from the thoracic and abdominal cavity. All the samples were immersed immediately in liquid nitrogen and stored at −80°C for further analysis.

### Histological Assay

Samples of liver were fixed with 10% formalin, embedded in paraffin, sectioned into 5 μm thickness, stained with hematoxylin/eosin (H&E) and finally analyzed by a NIKON ECLIPSE CI microscope (Nikon, Japan).

### Determination of Biochemical Parameters

Levels of fasted blood glucose (FBG) and serum uric acid (UA) were measured by commercial kits on a biochemical automatic analyzer. Hepatic triglyceride (TG), total cholesterol (TC), high-density lipoprotein (HDL-C), low-density lipoprotein (LDL-C), alanine aminotransferase (ALT). Hepatic tumor necrosis factor-α (TNF-α) and intestinal lipopolysaccharide (LPS) were quantified using enzyme-linked immunosorbent assay (Elisa) kits following the manufacturer’s instructions.

### 16S rRNA Gene Sequencing and Analysis

DNA for gut microbiota analysis was extracted from approximately 100 mg of faeces. DNA was amplified using a primer set targeting the V3+V4 region of 16S rDNA by carrying Barcode specific primer. The V3-V4 region of 16S rRNA was amplified using the primers 341F (CCTACGGGNGGCWGCAG) and 806R (GGACTACHVGGGTATCTAAT) by PCR. Raw reads were first confirmed using basic statistics. The processed pair-end reads were then merged using FLASH software (Version 1.2.11, United States) to generate representative complete sequences. The reads were qualitatively filtered in Trimmomatic0.33 and then clustered into 97% identity Operational Taxonomic Units (OTUs) using UCHIME4.2 to get effective tags. The effective tags were determined quantitatively and analyzed by Quanti-Fluorimeter and the Hiseq2500 system (Illumina, Inc., San Diego, CA, United States). LEfSe software (http://huttenhower.sph.harvard.edu/lefse/) is one way of statistical Beta diversity analysis. LEfSe was used for differential analysis inter groups. In this paper, LEfSe was shown to differential results. The selected differences were sorted by linear discriminant analysis (LDA) > 4.0.

### ^1^H-NMR Analysis of Hepatic Metobolites

The 100 mg liver tissue was homogenized by 600 μl of pre-cold CH_3_OH/H_2_O (2:1) on the ice. The extractive solution was vortexed for 30 s and centrifuged at 1600 rpm and 4°C for 10 min. The supernatant of 600 μl in a new tube was dried under nitrogen. Added with 600 μl of phosphate buffered saline (pH 7.4) containing 0.01% Sodium 3-trimethysilyl [2,2,3,3-d4] propionate (TSP-*d*_4_). The solution was vortexed and centrifuged again. The amount of 550 μl supernatant was pipetted and transferred into 5 mm NMR tube. The NMR tube was stored at 4°C for test.

^1^H-NMR spectrum was recorded on Bruker AVIII 600 MHz NMR (Bruker, Germany) spectrometer (proton NMR frequency at 600.13 MHz) equipped with ultra-low temperature probe by using a CPMG (CARR Purcell Meiboom Gill) pulse sequence. Key parameters of data acquisition were set as follows: the temperature at 300 K, spectrum width of 12,019.2 Hz, relaxation delay time of 4 s, 90° pulse width of 12 μs, acquisition time of 2.7263 s, scan times of 64, receiving gain 191 and sampling data point of 65,536.

Raw data were dealt with MestReNova software (Version 6.1.0, Spain). The process was carried out by phase correction and baseline correction before the methyl resonance of TSP-*d*_4_ was referenced to *δ* 0.000 ppm. The ^1^H NMR spectra from *δ* 0.7–9.0 ppm was bucketed into bins with an integral step of 0.001 ppm. The normalized integrity data were imported into Simca (Version 13.0, Sweden) to perform partial least squares discriminant analysis (PLS-DA). Differential metabolites were screened by Variable importance in the project (VIP) > 1 and *t*-test (*p* < *0.05*).

### Microarray Analysis of Hepatic Gene Expression

Total RNA was extracted from liver according to the test kit’s instruction and then treated with DNase I. Interrupt reagent was used to break mRNA into short segments. Six base random hexamers were used to synthesize cDNA based on the short segments of mRNA. The test kit was used to purify, repair, connect the test sequence and filter cDNA. The filtering conditions included: Removing the sequence that contains the connector, Sequencing with more than 5% N base removed and removing more than 50% of the sequences with base mass less than 10. The cDNA was amplified and enriched by PCR and QC tested by Agilent 2100 Bioanalyzer&ABI StepOnePlus Real-Time PCR System (Agilent Technologies Inc., California, United States). The data was sequenced by Illumina platform and the reads were compared by HISAT2 software (Version 2.0.5). Main parameter: no-mixed -I 100 -X 500—no-unal. In RNA SEQ analysis, we can use HTseq (Version 0.6.1) to further map the reads of the alignment genome to the gene exon region, and then count the number of reads on each gene alignment to estimate the gene expression level. Main parameters of HTseq: -s no -a 0 -t exon -m intersection-nonempty. Differential analysis was performed by DEGseq&DESeq platform among control, model and HME group. Differential genes were shown in volcano diagram and selected by absolute log_2_fold change (absolute FC) ≥ 0.5 and Q-value (or FDR) ≤ 0.01. The biological functions of differential genes were analyzed by KEGG (http://www.kegg.jp/). Differential pathway enrichments were screened by Q-value (or FDR) ≤ 0.01 and *t*-test (*p* < *0.05*).

### Statistical Analysis

The data were analyzed by GraphPad Prism software (Version 6.0.4, United States) and expressed as mean ± SD. One-way analysis of variance (ANOVA) with Dunnett test was employed to evaluate the significance of differences among animal groups. Differences were considered statistically significant at *p* < *0.05*. Partial least squares discriminant analysis (PLS-DA) was performed on Umetrics SIMCA (Version 13.0, Sweden). A value of *p or #*p* < 0.05, **p or ##*p* < 0.01, and ***p or ###*p* < 0.001 were considered statistically significant difference for all analyses.

## Results

### Chemical Profiling of Danggui-Shaoyao-San Extracts

Twenty-three kinds of chemical compounds from WE, HME and TME were identified by UPLC-Q/TOF-MS (Supplementary Figure 1), mainly including seven monoterpene glycosides, nine phthalides, three prototerpane triterpene glycosides, two cyclic peptides, two organic acids and adenosine. There were three kinds of differential MS fingerprints as shown in Supplementary Figure 2. The results about difference analysis (VIP > 1, *p* < *0.05*) showed that there were some differences in the chemical composition of the three extracts. There were ten different substances between WE group and HME group, which were gallic acid, ferulic acid, albiflorin, alisol B-23-acetate, senkyunolide I, lactiflorin, mudanpioside I, Senkyunolide A, *Z*-ligustilide and cnidilide A. Ten different substances were screened out between WE group and TME group, including gallic acid, albiflorin, isomaltopaeoniflorin, alisol B-23-acetate, mudanpioside I, senkyunolide A, *Z*-ligustilide, cnidilideA, alisol C-23-acetate, and tokinolide B. Six different substances were screened out between HME group and TME group, including atractylenolideI, alisol B-23-acetate, senkyunolide I, lactiflorin, senkyunolide A, and *Z*-ligustilide. The PLS-DA analysis indicated the obvious classification and clustar among three extracts (Supplementary Figure 3). Seven chemical compounds were screened out between WE and HME/TME. They were separately gallic acid, paeoniflorin, alisol B-23-acetate, mudanpioside I, senkyunolide A, Z-ligustilide and cnidilide A. Results indicated that the mixture of methanol and water or methanol upregulated the content of seven compounds as shown in Supplementary Figure 4. It has been reported recently that phthalides (cnidilide, ligustilide, senkyunolide) from Chuanxiong have anti-inflammatory,vascellum protection, anti-thrombotic, anti-oxidant, anti-hypertensive properties (Yue et al., 2018; Ningsih et al., 2020). Cnidilide is found to lower LPS, inflammatory factors such as TNF-α, interleukin-1beta (IL-1β) and interleukin-6 (IL-6) (Lee et al., 2016). Gallic acid is well known not only as antioxidant capacity but also for neuroprotective elements (Daglia et al., 2014). We concluded that different solvents can yield different extracts with different compounds, which may influence the MetS symptoms by these differential compounds.

### Danggui-Shaoyao-San Effects on Liver Histopathology and Biochemical Parameters

**FIGURE 1:**
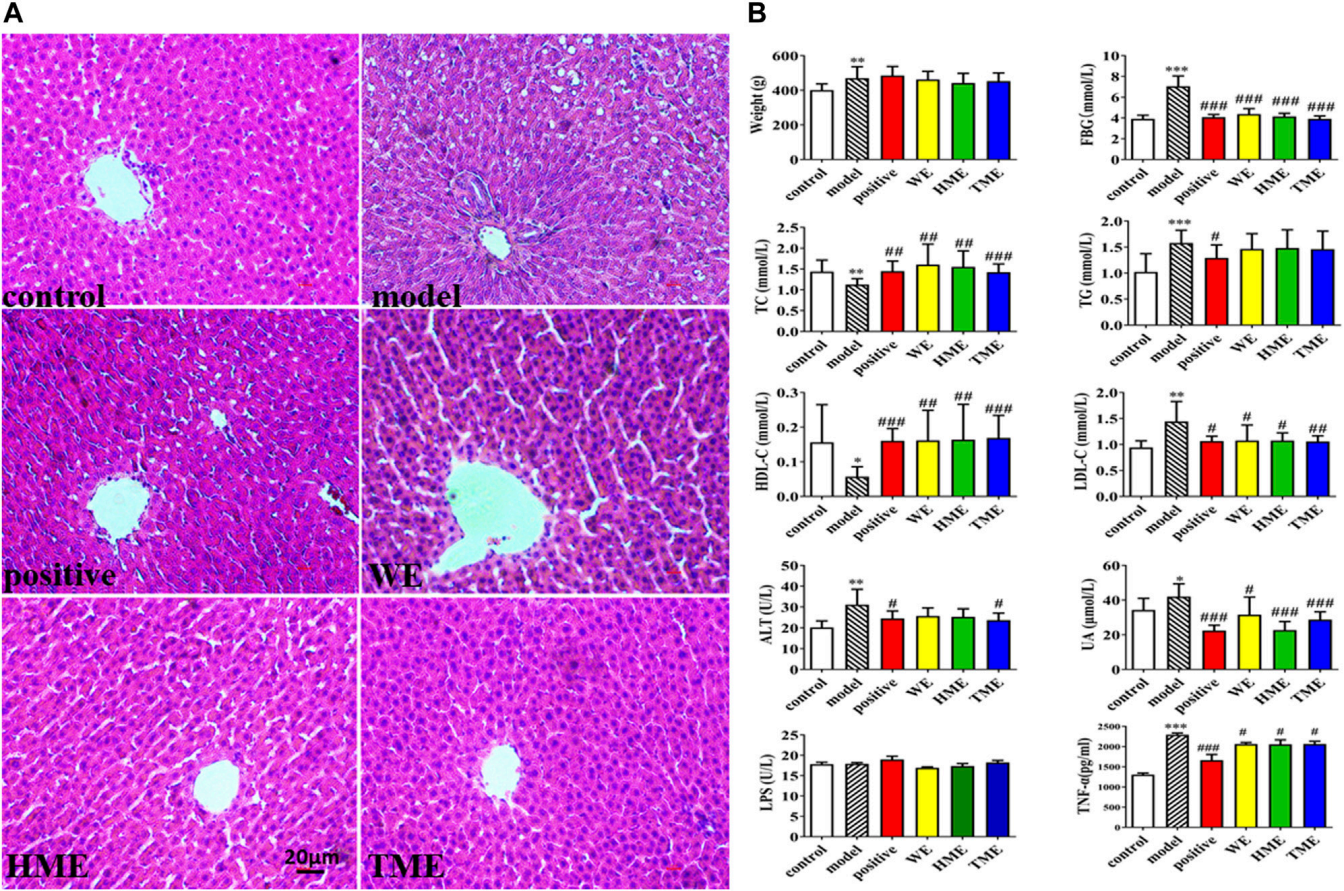
Histopathological assessment in liver (H&E stain) and biochemical assessments in serum or liver. **(A)** pathological paraffin section (100X) **(B)** Levels of fasted blood glucose (FBG), serum uric acid (UA), hepatic triglyceride (TG), total cholesterol (TC), high density lipoprotein (HDL-C), low density lipoprotein (LDL-C), alanine aminotransferase (ALT), tumor necrosis factor-α (TNF-α) and intestinal levels of lipopolysaccharide (LPS). All values are mean ± SD (*n* = 10). *p < *0.05*, **p < *0.01*, ***p < *0.001* between model group and control group, #*p* < *0.05*, ##*p* < *0.01*, ###*p* < *0.001* between model group and WE/HME/TME group.

Pathological study and biomarkers can be used to preliminarily evaluate which extracts can influence the symptoms of MetS. The liver pathological sections of rats in each group were observed under 100x high power microscope (Figure 1A). There were tensile vacuoles with different sizes and density in the cytoplasm of hepatocytes in the model group, compared with the control group. According to the scale bar, the diameters of the vacuoles were about 2–15 μm. These vacuoles were supposed to generate due to lipid homeostasis in the liver. In different treatment groups, there was less vacuole in quantity and size. The results of H&E staining indicated that DSS protected liver tissue. Compared with the control group, FBG, body weight, UA and hepatic TG, LDL-C, ALT statistically up-regulated in the model group (*p* < *0.05*), while hepatic TC and HDL-C statistically down-regulated (*p < 0.05*, Figure 1B). It indicated that metabolic syndrome and hepatic injury of rats in the model group were obvious. Enteral LPS and hepatic TNF-α were also detected, and the results showed a significant increase of TNF-α (*p* < *0.001*) but no obvious change of LPS in the model group (Figure 1B). It was found that FBG, TNF-α, LDL-C and UA significantly decreased (all *p < 0.05*) and TC and HDL-C increased (all *p < 0.05*) after the use of pioglitazone or three extracts. ALT had got regulation (*p < 0.05*) after the intervention of pioglitazone or TME. Although pioglitazone or different extracts had no obvious effect on the improvement of body weight, body weight had the down tendency in HME or TME group, especial HME group. The statistical biomarkers, including TC, HDL-C, LDL-C, ALT and UA, revealed that HME or TME had stronger protective actions for liver functions and regulated the MetS better than WE.

### Changes in Gut Microbiota Structure

**FIGURE 2:**
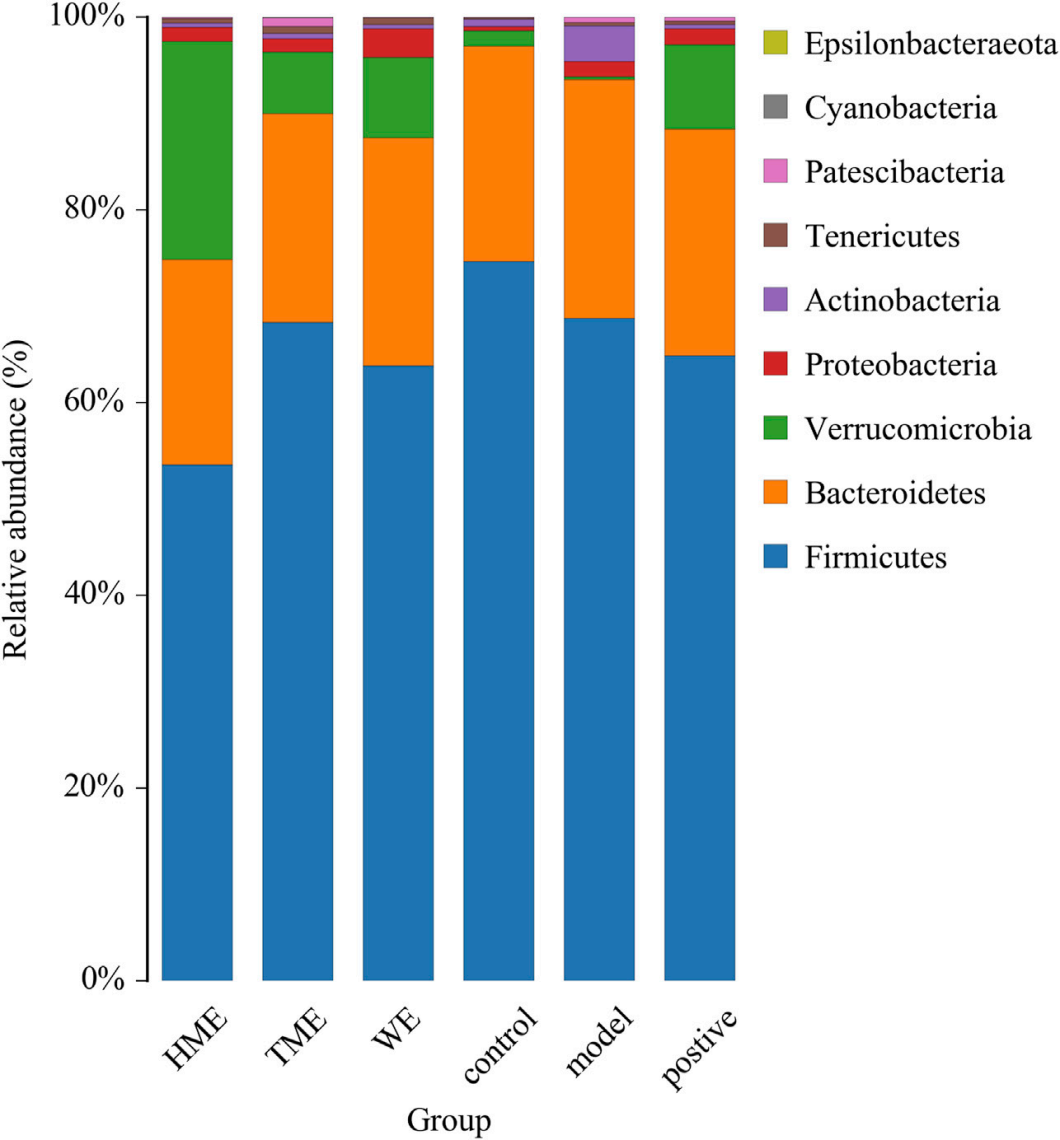
Histogram of mean value of intestinal microflora enrichment in rats at phylum level in each group.

Gut microbiota plays an important role in evaluating all kinds of organism, balancing the unhealthy environment in our bodies and keeping us healthy. We calculated the OUT expression of each sample at all kinds of classification level, especially at the level of phylum and genus to find which extract can influence the kinds and enrichment in intestinal microbiota to alleviate abnormal symptoms about MetS. As shown in Figure 2 dominant bacteria were *Firmicutes*, *Bacteroides*, *Verrucomicrobia* in sequence at the phylum level. The relative abundance of *Firmicutes* had its downward trend in the WE, HME, TME group, while *Bacteroides* and its genus almost kept constant. In addition, *Verrucomicrobia* in the different treatment groups had a growing tendency, while it was almost absent in the model group. It is reported that the change of relative abundance of *Firmicutes* is positively related to the occurrence of metabolic diseases such as obesity (Koliada et al., 2017). It has also been reported that *Akkermansia*, belonging to *Verrucomicrobia,* is negatively related to obesity, and the higher the relative abundance in the intestine, the less obesity the host appears (Brahe et al., 2015; Dao et al., 2016; Derrien et al., 2017).

**FIGURE 3:**
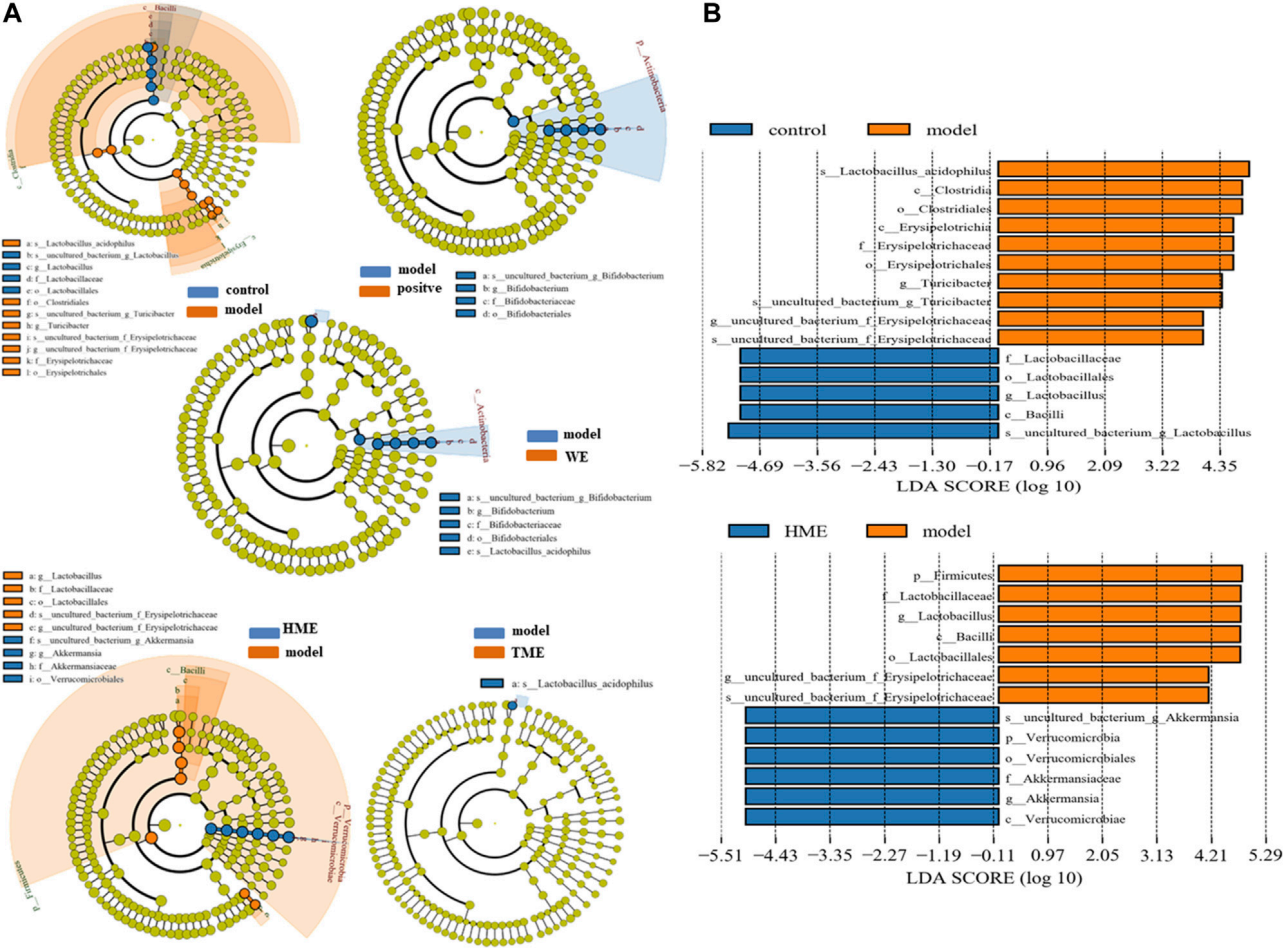
LEfSe results of species differences in intestinal microflora enrichment in rats between different groups. **(A)** Comparative analysis by cladogram pattern between control and model group or model and WE/HME/TME group. **(B)** Comparative analysis by LDA histogram only in control/model or model/HME contrast (LDA > 4.0).

LEfSe was used to analyze the difference of gut microbes. The result at the genus level was expressed by Cladogram pattern or LDA histogram (LDA > 4.0). From the results, we concluded that there was more differential gut microbiota from HME group (Figure 3A) as compared with the control group. The differences from the model group was mainly reflected in the up-regulation of the relative abundance of *Turicibacter* and *Erysipelotrichaceae*, and the down-regulation of *Lactobacillus* (LDA > 4.0, Figure 3B) by LDA histogram analysis. The HME group contained the richest gut microbes including downregulated *Lactobacillus* and *Erysipelotrichaceae*, and the notably upregulated *Akkermansia* (LDA > 4.0). It is reported that *Akkermansia* is beneficial bacteria to reduce obesity (Everard et al., 2013; Shin et al., 2014) and *Erysipelotrichaceae* is harmful by increasing intestinal inflammation (Wu et al., 2018). Based on the changes in gut microbes, it can be concluded that HME can play a more diversifying and beneficial effect on regulating gut microbiota structure than WE and TME. Based on all the results, further studies on metabolomic and transcriptomic changes in the liver were carried only by the HME treatment.

### Metabolomic Changes in Liver

**FIGURE 4:**
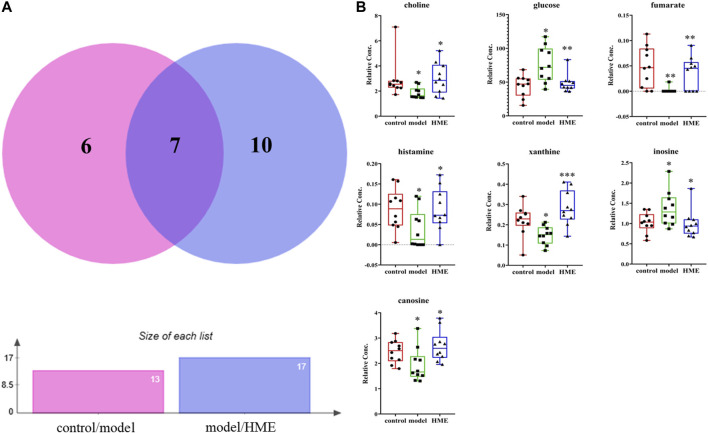
Differential metabolites in common in rat liver between different groups. **(A)** Differential metabolites changes in common by Wayne analysis in pie chart between control/model or model/HME contrast. **(B)** Individual relative content changes of differential metabolites in common by histogram pattern (differential screening criterion: VIP > 1 and *p < *0.05*, **p < *0.01*, ***p < *0.001*).

The changes of metabolites in the liver can show whether the hepatic functions or metabolic pathways have been influenced by fructose. According to NMR data, a variety of metabolites in the liver were identified, mainly including amino acids, organic acids, alkaloids, sugars and nucleotides. Choose VIP > 1, *p < 0.05* as screening criteria to screen the differential metabolites between different groups. Thirteen differential metabolites were identified between the control group and model group, which were 3-hydroxybutyrate, alanine, carnosine, choline, glycine, glucose, fumarate, anserine, tyramine, histamine, xanthine, inosine, formate, respectively. Seventeen differential metabolites were identified between the model group and HME group, which were respectively isoleucine, leucine, valine, lactate, acetate, succinate, carnosine, cadaverine, creatine, choline, glutamine, glucose, ADP + ATP, fumarate, histamine, xanthine and inosine. There were seven shared metabolites by Venny analysis (http://jvenn.toulouse.inra.fr/app/index.html) between control/model and model/HME, including carnosine, choline, glucose, fumarate, histamine, xanthine, inosine (Figure 4). From thirteen differential metabolites obtained between the control and model group, we concluded that there were certain metabolic disorders in the model group. By the intervention of DSS, seven differential metabolites can be regulated back in the same changed metabolic routes by fructose. The other ten differential metabolites between the model and HME group indicated that DSS could influence the new metabolic pathways. The results showed that DSS played an effective role in improving the MetS.

### Transcriptomic Changes in Liver

The differential transcripts between different groups were screened at absolute FC ≥ 0.5, Q-value and FDR ≤ 0.01. By analyzing the metabolic pathway by differential gene expressions, we determined which pathways had been disorganized by fructose in the model group and which were regulated back after DSS. Compared with the control group, 422 transcripts were up-regulated, 377 transcripts were down-regulated and 18,867 transcripts were the same in the model group. Compared with the model group, there were 132 up-regulated transcripts, 218 down-regulated transcripts and 19,336 transcripts were the same in HME group. The results showed that high fructose-fed did change the hepatic gene expressions of the liver gene in normal rats, while to a certain extent, DSS did alleviate the abnormal situation of hepatic transcripts in the model group.

**FIGURE 5:**
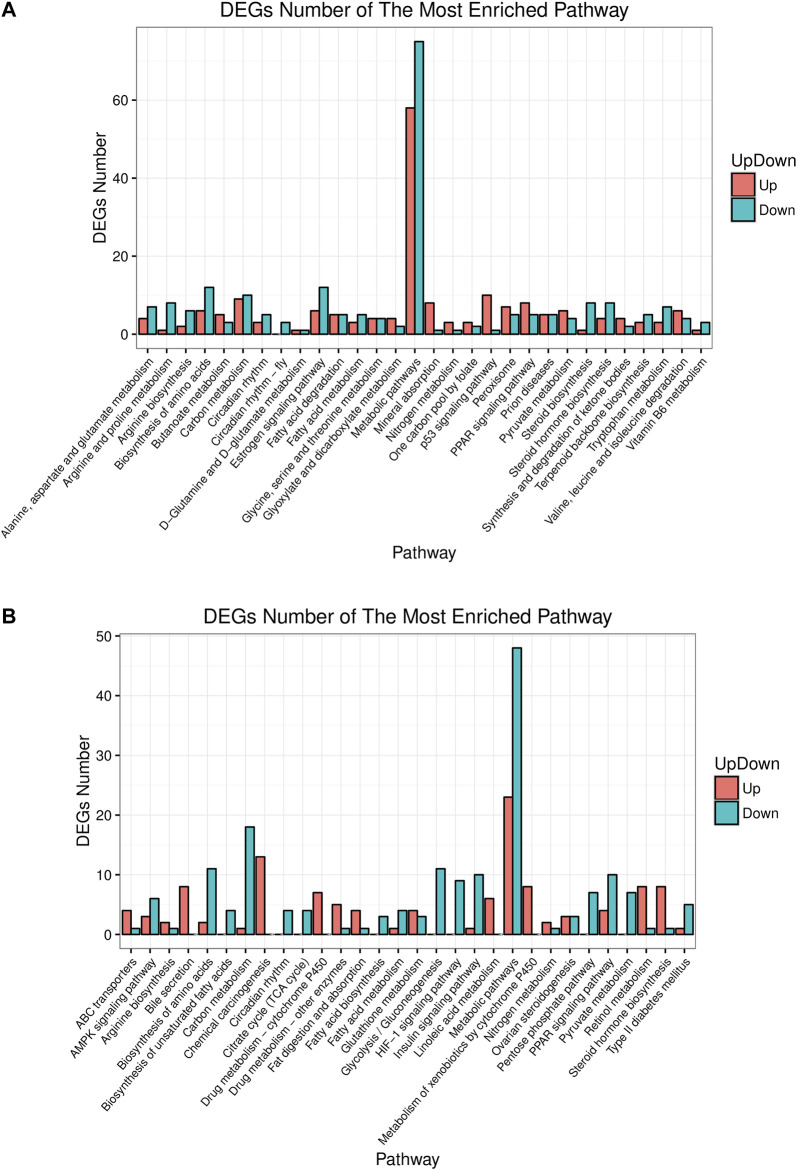
Up or down about differential gene experessions in top 30 enrichment pathways in rat liver between control/model or model/HME. **(A)** Comparative analysis by histogram pattern between control group and model group. **(B)** Comparative analysis by histogram pattern between model group and HME group. (differential screening criterion: absolute FC ≥ 0.5, Q-value or FDR ≤ 0.01).

Pathway analysis helps understand the biological function of genes. In this study, KEGG analyzed how the gene expressions were enriched between different groups. The top 30 differential pathways at the Level 3 were analyzed between the control and model group (Figure 5A). Another result was got between the model and HME group as shown in Figure 5B. The pathway with the most enriched differential genes was focused on the metabolic pathway. Compared with the control group, fifty eight differential genes in the model group were upregulated and seventy five genes were downregulated in the metabolic pathway. Compared with the model group, twenty three differential genes in the HME group were upregulated and forty eight were downregulated. Other ten shared pathways were arginine biosynthesis, steroid hormone biosynthesis, biosynthesis of amino acids, carbon metabolism, circadian rhythm, fatty acid metabolism, metabolic pathways, nitrogen metabolism, PPAR signalling pathway and pyruvate metabolism. In these pathways, some were related to the carbohydrate metabolism. Hepatic differential metabolites such as glucose and fumarate also showed the correlation with carbohydrate metabolism. The genes of HSD family, such as hydroxysteroid 17β-dehydrogenase 7 (HSD17β7) and hydroxysteroid 17β-dehydrogenase 13 (HSD17β13), existed in the pathway of steroid hormone biosynthesis. They regulated blood lipid and were related to TC and TG metabolites. These results showed that fructose could induce differential gene changes and pathway discordances and DSS could regulate back these abnormalities of carbohydrate, lipid and amino acids at the Level 2 of the KEGG pathway.

## Discussion

### The Relationship Between Metabolic Syndrome in Fructose-Fed Rat Modeling and Insulin Resistance

A large body of recent literature has shown that excess fructose comsumption can induce MetS (Li et al., 2018). 15% of fructose is similar to American human soft drinks (Jürgens et al., 2005).Many researchers have reported that 10–20% fructose for 4–8 weeks could induce MetS modeling or one of MetS symptoms in rat or mice (Jürgens et al., 2005; De Moura et al., 2008; Zhang et al., 2011). The modeling conditions are similar with our research. IR is reported as the mechanism of MetS and chronic fructose ingestion, but not acute fructose feedings can lead to hyperinsulinemia and insulin resistance (Haas et al., 2012; Pan and Kong, 2018). Thus, we reviewed some literature on mechanism how IR develops high fructose-induced MetS.

Fructose directly reaches the hepatocytes after being absorbed from the intestine via portal vein. The metabolic pathway of most of fructose is the firs-pass metabolism in the liver (Softic et al., 2016). Fructolysis is much faster than glycolysis in the hepatocytes for bypassing the step of phosphofructokinase (Pan and Kong, 2018), which usually limits the metabolic speed. Limitless fructolysis can produce high level of *de novo* lipogenesis (DNL) and UA metabolites (Pan and Kong, 2018). They are unique features of fructolysis and high level of them can induce insulin resistance. After entering the hetatocytes, fructolsis is catalyzed by ketohexokinase (KHK) and aldolase B (ALDOB), which are two specific enzymes and highly expressed in the hepatocytes (Pan and Kong, 2018). In the first-pass metabolism, fructolysis happens quickly with comsuming more ATP by KHK (Asipu et al., 2003; Softic et al., 2016) for fructose phosphoration. This process converts fructose into fructose-1 phosphate (F-1-P) and finally produces plenty of UA (Nakagawa et al., 2006). Excessive UA directly inhibits insulin signaling not only in hepatocytes (Zhu et al., 2014), but also in endothelial cells (Choi et al., 2014) and cardiomy-ocytes *in vitro* (Zhi et al., 2016). The inhibition leads to insulin resistance in potassiumoxonate-induced hyperuricemia mice (Zhu et al., 2014). The second catalytic enzyme is ALDOB, which can help part of F-1-P directly converted into dihydroxyacetone phosphate (DHAP) and indirectly into glycerol-3-phosphate (GA3P) (Nomura and Yamanouchi, 2012). DHAP and GA3P are catabolized for DNL via glycolytic pathway by intermediate acetyl-CoA, to be used in tricarboxylic acid (TCA) cycle (also known as citric acid cycle), for energy production (Softic et al., 2016). High level of DNL leads to alteration of TG, TC and free fatty acid (FFA) and other lipid metabolites and induces insulin resistance under high fructose condition (Huang et al., 2017).

Oxidative stress and inflammation play causal roles in insulin resistance (Pan and Kong, 2018). More and more evidence demonstrates that they impair insulin signaling to induce insulin resistance in high fructose-induced MetS (Bettaieb et al., 2015; Porto et al., 2015). High level of Reactive oxygen species (ROS) or disability of anti-oxidation stimulated by FFAs, especially saturated FFAs, induces oxidative stress (Inoguchi et al., 2000). Lipid metabolites, such as FFAs, TG, diacylglycerol (DAG), and ceramides, can activate pro-inflammatory kinases, thus impair insulin signaling in hepatic or extrahepatic tissues (Xu et al., 2003; Choi et al., 2008). Pro-inflammatory kinases relate to protein kinase C (PKC), c-jun N-terminal kinase (JNK) and inhibitor of nuclear factor kappa B (NF-*k*B) (I*k*B) kinase (IKK) (Bergman and Ader, 2000). On the other hand, fructose-induced UA metabolite also causes ROS production in hepatic (Lanaspa et al., 2012) and extrahepatic tissues (Sautin et al., 2007; Hu et al., 2009; Lanaspa et al., 2012; Cirillo et al., 2015; Madlala et al., 2016) and activates inflammatory response by induction of IL-1β, TNF-α, transforming growth factor (TGF)-β1 and monocyte chemotactic protein1 (Umekawa et al., 2003; Palanisamy et al., 2011; Chen et al., 2013) Finally these inflammatory factors also induce IR. Therefore, DNL and UA are dangerous factors induced by chronic fructose ingestion. IR, whose underlying common mechanisms are oxidative stress and inflammation, is the key pathological mechnism in MetS.

### The Mechanism of Regulating Blood Lipid by Danggui-Shaoyao-San in Metabolic Syndrome Rats

Compared with the control group, part of biomarkers, body weights, pathological sections in the model group have shown that there was a disorder in the hepatic functions and carbohydrate metabolism. This indicated the occurrence of nonalcoholic fatty liver disease (NAFLD). NAFLD is closely related to MetS. It is reported that inducing NAFLD is related to the rapid increase of hepatic TNF-α (Wang et al., 2012). HSD17β13 is also identified as a pathogenic protein in the development of NAFLD (Kampf et al., 2014; Su et al., 2014). In general, HSD family takes part in several kinds of metabolism, but the HSD17β13 mainly takes part in lipid metabolism (Fujimoto et al., 2004; Moeller and Adamski, 2009; Marchais-Oberwinkler et al., 2011; Su et al., 2014).The upgrade of TG is reported due to overexpression of HSD17β13. In our study, HSD17β13 (Q ≤ 0.01) (Supplementary Table 2) and TNF-α (*p < 0.05*) were both found upregulated.TG (Figure 1B) had its marked upgraded trend (*p < 0.001*). On contrary, all the results had their downgrade without statistics (*p > 0.05*) after the cure with DSS. All the data conformed to the conclusion and the literature that TG is positively and directly related to HSD17β13 and TNF-α (Su et al., 2014).

It is reported that HSD17β7 in a rat is beneficial to increase TC (Matsuoka et al., 2020). In this gene study, as compared with the control group, HSD17β7 (Supplementary Table 2) statistically decreased (Q ≤ 0.01) in the model group. TC (Figure 1B) in the model group abnormally decreased (*p < 0.01*). Conversely, TC (*p < 0.01*) and HSD17β7 (Q ≤ 0.01) increased markedly in the HME group. This showed that there was a positive relationship between TC and HSD17β7. It is reported that TC can produce bile acid after a series of metabolism. Bile acid is the main way to excrete cholesterol (Iritani et al., 1982). Most bile acids exist as conjugated bile salts. A small part of bile salts are decomposed into insoluble free bile acids by the bile salt hydrolytic enzymes secreted by the *Lactobacillus* in the intestine (Gilliland et al., 1985; Rasic et al., 1992; Klaver and van der Meer, 1993). These free bile acids can be excreted along with faeces *in vitro*. The lowered bile acid salts *in vivo* accelerated the transformation from TC to bile acid. The excretion pathway of bile acids can be the regulated targeting to lower blood lipid. *Lactobacillus* (Supplementary Table 1) decreased (LDA > 4.0) in the model group compared with the control group. Compared with the model group, *Lactobacillus* was continuously decreased in the HME group. TC finally increased (*p < 0.01*) and was even higher than the control group in the HME group. Based on the facts, we concluded that the significant up-regulation of TC might be possibly due to the double effect from the upgrade of HSD17β7 and the downgrade of *Lactobacillus*.

### The Mechanism of Anti-Inflammation by Danggui-Shaoyao-San in Metabolic Syndrome Rats

A high level of DNL and UA can result in insulin resistance, which is the key pathological event in developing MetS. It happens mostly through oxidative stress and inflammation (Nakagawa et al., 2006; Schwarz et al., 2017; Pan and Kong, 2018). Fructose-induced UA can trigger an inflammatory response. ROS induction can induce inflammatory factors such as interleukin, tumour necrosis factor and transforming growth factor (Umekawa et al., 2003; Palanisamy et al., 2011; Chen et al., 2013; Pan and Kong, 2018). In comparison with the control group, UA (Figure 1B) in the model group significantly increased (*p < 0.001* indicating an inflammatory reaction. After DSS treatment, UA decreased significantly (*p* < *0.001*), indicating an anti-inflammatory effect of DSS. There are many kinds of inflammation genes to perform the anti-inflammation, including the NOD-like receptor (NLR) family, TNF family and IL family (Huang et al., 2019). Some part of inflammation genes overexpresses when inflammation occurs. In this study, NOD-like receptor pyrin containing 12 (NLRP12) (Q ≤ 0.01) and TNF-α (*p* < *0.05*) upgraded markedly in the model group, while IL-33 (Q ≤ 0.01) downgraded statistically (Supplementary Table 2). After the cure of DSS, although IL-33 upgraded markedly (Q ≤ 0.01), the level of IL-33 was still lower than that in the control group. TNF-α was regulated back to a normal level statistically (*p < 0.05*). It revealed that the inflammation was being alleviated. NLRP12 was gradually increased using DSS but had no significant difference. All the evidence suggested that there was a possible relationship between NLRP12, TNF-α, IL-33 and DSS’s anti-inflammatory mechanism. Inflammation occurs due to the changes from genes, intestinal flora and its metabolites. It is reported that *Erysipelotrichaceae*, *Turicibacter* and others are related to the elevation of TNF levels, IL levels in the Muc2 deficient mice with colitis (Wu et al., 2018). *Erysipelotrichaceae* (Supplementary Table 1) markedly upgraded in model group (LDA > 4.0), accompanied with the increase of TNF-α. On the contrary *Erysipelotrichaceae* (LDA > 4.0) and TNF-α (*p < 0.05*) synchronously markedly downgraded after the cure of DSS. This result further demonstrated that *Erysipelotrichaceae* can be directly related to inflammation and DSS worked as an anti-inflammatory by reducing the enrichment of *Erysipelotrichaceae* and regulating back TNF-α level.

One of the characteristics of MetS is IR, which is an inflammatory disease (Donath and Shoelson, 2011). It is also reported that intestinal flora ferments amino acids to produce acetate, butyrate and formate which belong to short-chain fatty acids salt with the anti-inflammation effect (Davila et al., 2013). For example, *Lachnospiraceae* family can produce butyrate (Costantini et al., 2017). *Akkermansia* is another new and strongly beneficial bacterium except for *Lactobacillus* which is resistant to human obesity and MetS (Brahe et al., 2015). It exists in less quantity in the intestine of an obese human and plays an important role in maintaining intestinal health and integrity. It releases acetate through the degradation of mucin to regulate membrane permeability (Dao et al., 2016). Results demonstrated that all kinds of intestinal flora can play a role in different ways about anti-inflammation. In comparison with the control group, *Lachnospiraceae_NK4A136_group* (Supplementary Table 1) increased in the model group without statistics (LDA < 4.0). *Akkermansia* (Supplementary Table 1) almost absent statistically (LDA > 4.0). Hepatic metabolites-acetate (Supplementary Table 3) increased without statistics (LDA < 4.0). After the treatment of DSS, the relative abundance of *Lachnospiraceae_NK4A136_group* (Supplementary Table 1) slightly downgraded with no statistics. But acetate and formate (Supplementary Table 3) both increased markedly (VIP > 1, *p < 0.05*.0) after the cure of DSS. The reason for the change could be due to the very rapid increase of *Akkermansia* (LDA > 4.0) and there was a strong positive correlation. The efficient ingredient in DSS had the function of anti-inflammation. DSS may contribute to the increase of *Akkermansia* and its metabolite-short-chain fatty acids salt to regulate membrane permeability and anti-inflammation.

### The Mechanism of Reducing the Blood Glucose by Danggui-Shaoyao-San in Metabolic Syndrome Rats

The level of amino acid in the blood can predict the occurrence of diabetes as amino acid is an important material of gluconeogenesis and it leads to the increase of blood glucose (Chen et al., 2016). Fumarate is reported to be a crucial enzyme in TCA cyclic reaction and has a catalytic role in amino acid metabolism (Suzuki et al., 1989; Arts et al., 2016). The higher the amino acid metabolic speed, the lower is the concentration of amino acids in the blood. Compared with the control group, the differential down-regulation of fumarate (Supplementary Table 3) in the model group (VIP > 1, *p < 0.05*) slowed down the speed of amino acid metabolism, which led to the significant up-regulation of glycine and alanine (Supplementary Table 3) in liver and glucose in the blood (VIP > 1, *p < 0.05*). Compared with model group, HME group regulated back the concentration of fumarate (VIP >1, *p < 0.05*). It led to decreasing the content of glycine and alanine, slowing down the gluconeogenesis, and significantly reducing the glucose levels (VIP >1, *p < 0.05*) (Supplementary Table 3). Besides glycine and alanine, there were other branched amino acids (BCAAs) in the blood which were valine, leucine and isoleucine. In this study, BCAAs (Supplementary Table 3) in the different groups were relatively low. Glycine and alanine contributed more to gluconeogenesis in the aspect of both content and changing amplitude and played major roles in the decreasing of glucose level in the blood. Even though we should discuss the relationship between BCAAs and *Prevotella_9* (Supplementary Table 1), the relationship is also important. Other studies indicated that the increase of BCAAs in the blood of mice fed by *Prevotella_9* resulted in IR, glucose intolerance and Type 2 diabetes mellitus (T2DM). The increase of BCAAs in blood has been used as a marker of IR and T2DM (Lynch and Adams, 2014; Pedersen et al., 2016). Other literature has reported that compared with the Normal group, *Prevotella_9* in T2DM model group increases significantly, accompanied by the increase of the metabolite-BCAAs in the blood (Pedersen et al., 2016; Yue et al., 2018). Results obtained demonstrated that increase of BCAAs is positively correlated with *Prevotella_9*. By analyzing the data of this study, we found that compared with the control group, the BCAAs of the model and HME groups were gradually increasing with the increase of relative abundance of *Prevotella_9*. It indicated that the trend of this experiment was consistent with the literature and there was a strong positive correlation between BCAAs and *Prevotella_9*.

Lactate is the product of anaerobic fermentation of glucose under anaerobic conditions. The increase of lactate shows the growth of Glycolysis and gluconeogenesis accompanied by more glucose in the blood. In this study, compared with the control group, the increase of lactate in the blood in the model group indicated that gluconeogenesis generated more glucose. On the contrary, compared with the model group, HME statistically regulated the lactate (Supplementary Table 3) and FBG (Figure 1B) (VIP > 1, *p < 0.05*). There were a lot of changes in the intestinal flora. These changes included *Lactobacillus* and *Turicibacter* (Supplementary Table 1) which produced lactate (Li et al., 2017). Compared with the control group, *Lactobacillus* producing lactate markedly came down (LDA > 4.0) and *Turicibacter* markedly upgraded (LDA > 4.0) in the model group. This suggested that *Turicibacter* may be the key factor to producing lactate. Compared with the model group, there was marked downgrade of lactate (VIP > 1, *p < 0.05*) and a marked downgrade of *Lactobacillus* (LDA > 4.0) after the cure with DSS. *Turicibacter* had a slight upgrade without statistics after the cure with DSS. In this aspect, we concluded that the change of lactate in blood was mainly due to the downgrade of *Lactobacillus*. All the results showed that the change of lactate in blood was not completely induced by *Lactobacillus*, also by other bacteria which can produce lactate, such as *Turicibacter*. In conclusion, the combined action of host differential metabolites and intestinal flora can play a role in regulating the abnormal blood glucose in MetS.

## Conclusion

**FIGURE 6:**
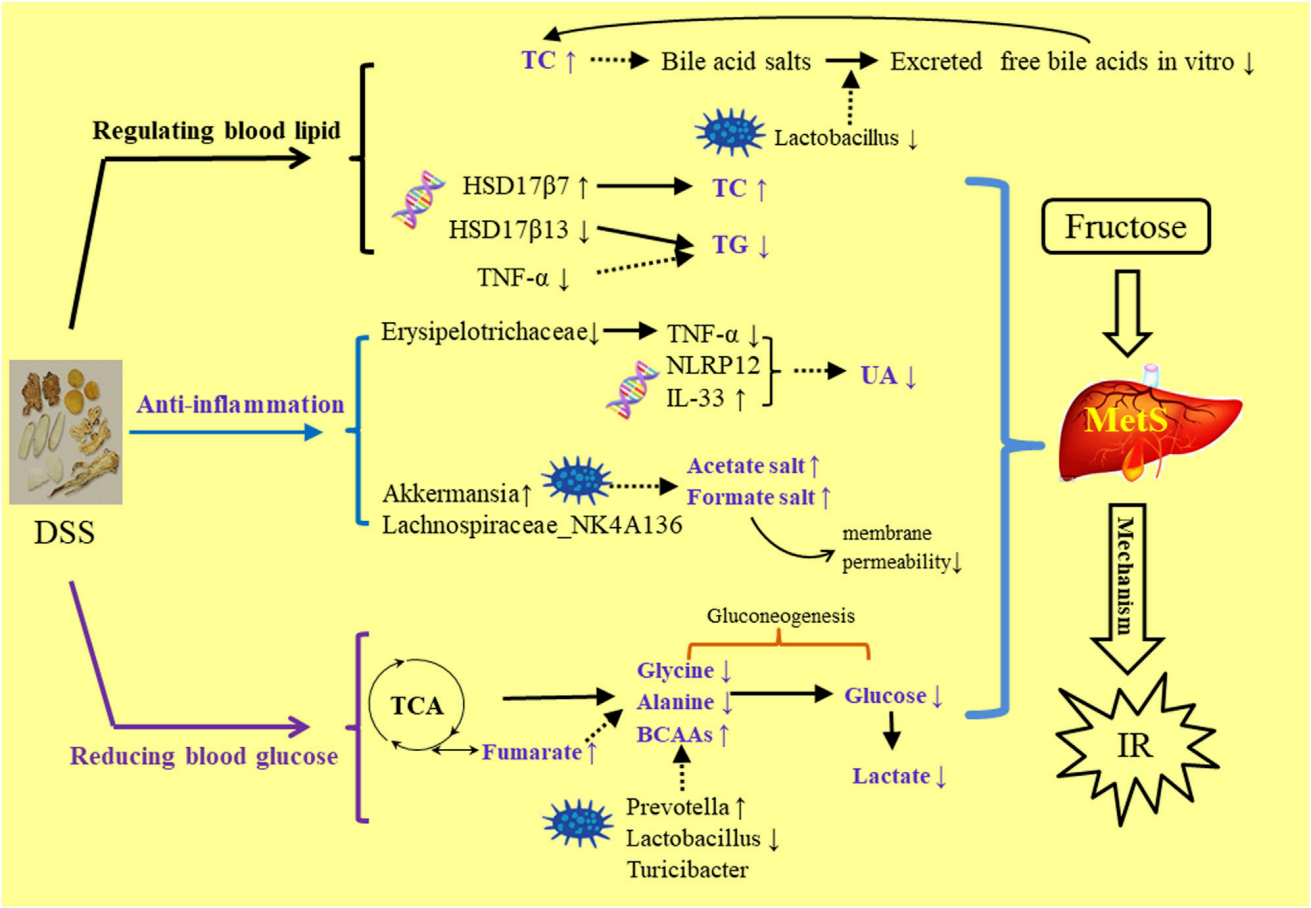
The mechanism study of DSS’s regulating MetS symptoms in three aspects of blood lipid, anti-inflammation, blood gluctose.

DSS is a traditional prescription with bolstering blood cycle and alleviating stasis due to blood stasis and deficiency of spleen Qi. This study was the first to report about alleviating the MetS symptoms. In the comparison of the three extractive solvents, it was found that HME or TME had seven different chemical components with higher content than WE. These compounds have been recorded in the aspects of vascellum protection, anti-inflammatory, anti-thrombotic, anti-oxidant effect. In the HE-staining pharmacological section, liver biochemical parameters, 16S rRNA intestinal flora research, it was preliminarily concluded that the effect of 50% methanol extract was more prominent to be used to MetS. By the analysis of relationships in HME group between different liver metabolites, liver transcriptional genome and intestinal flora, we can understand how DSS played a role in reducing blood lipid, anti-inflammatory and hypoglycemic (Figure 6). The results laid a foundation for the further exploration of clinical application and effective mechanism of DSS in MetS in the future. This study can give some guidance on more valid components of traditional Chinese medicine in the aspect of improving gut microbia dysbiosis and hepatic lipid homeostasis in fructose-fed rats.

## Data Availability

The datasets presented in this study can be found in article, Supplementary Material and online repository: Gene expression omnibus (GEO) from NCBI. The accession number can be found below: GEO, GSE176572.
