# Supplementary material for: Danggui-Shaoyao-San Improves Gut Microbia Dysbiosis and Hepatic Lipid Homeostasis in Fructose-Fed Rats
Source: Front Pharmacol. 2021 Jul 13;12:671708. doi: 10.3389/fphar.2021.671708 (PMC8313808; doi:10.3389/fphar.2021.671708)
Supplement: Supplementary file 1 [file DataSheet1.docx]

Supplemental Information

**Danggui-Shaoyao-San Improves Gut Microbia Dysbiosis and Hepatic Lipid Homeostasis in Fructose-Fed Rats**

Jing Yin^1^, Jiaxi Lu^1^, Peng Lei^1^, Mingshuai He^1^, Shengjie Huang^1^, JialinLv^1^, Yan Zhu^1^, Zhidong Liu^1^, MiaomiaoJiang^1, 2,^ *

^1^ State Key Laboratory of Component-based Chinese Medicine, Tianjin University of Traditional Chinese Medicine, Tianjin 301617, China

^2^ Department of Pharmacy, Institute of Traditional Chinese Medicine, Tianjin University of Traditional Chinese Medicine, Tianjin 301617, China

Jing Yin [happy_yin@126.com](mailto:happy_yin@126.com)

Jiaxi Lu [18712852757@163.com](mailto:18712852757@163.com)

Peng Lei [leipengcn@163.com](mailto:leipengcn@163.com)

Mingshuai He [h1115049914@163.com](mailto:h1115049914@163.com)

Shengjie Huang [huang962021@sina.com](mailto:huang962021@sina.com)

Jialin Lv [ljl_bailey@163.com](mailto:ljl_bailey@163.com)

Yan Zhu [yanzhuharvard@gmail.com](mailto:yanzhuharvard@gmail.com)

Zhidong Liu [lonerliuzd@163.com](mailto:lonerliuzd@163.com)

* Corresponding author at: Tianjin University of Traditional Chinese Medicine, 10 Poyanghu Road, West Area, Tuanbo New Town, Jinghai District, Tianjin, P. R. China, 301617.

E-mail: [miaomiaojiang@tjtcm.edu.cn](mailto:miaomiaojiang@tjtcm.edu.cn)





**Figure S1. The structures of all the identified chemical compounds.**

**
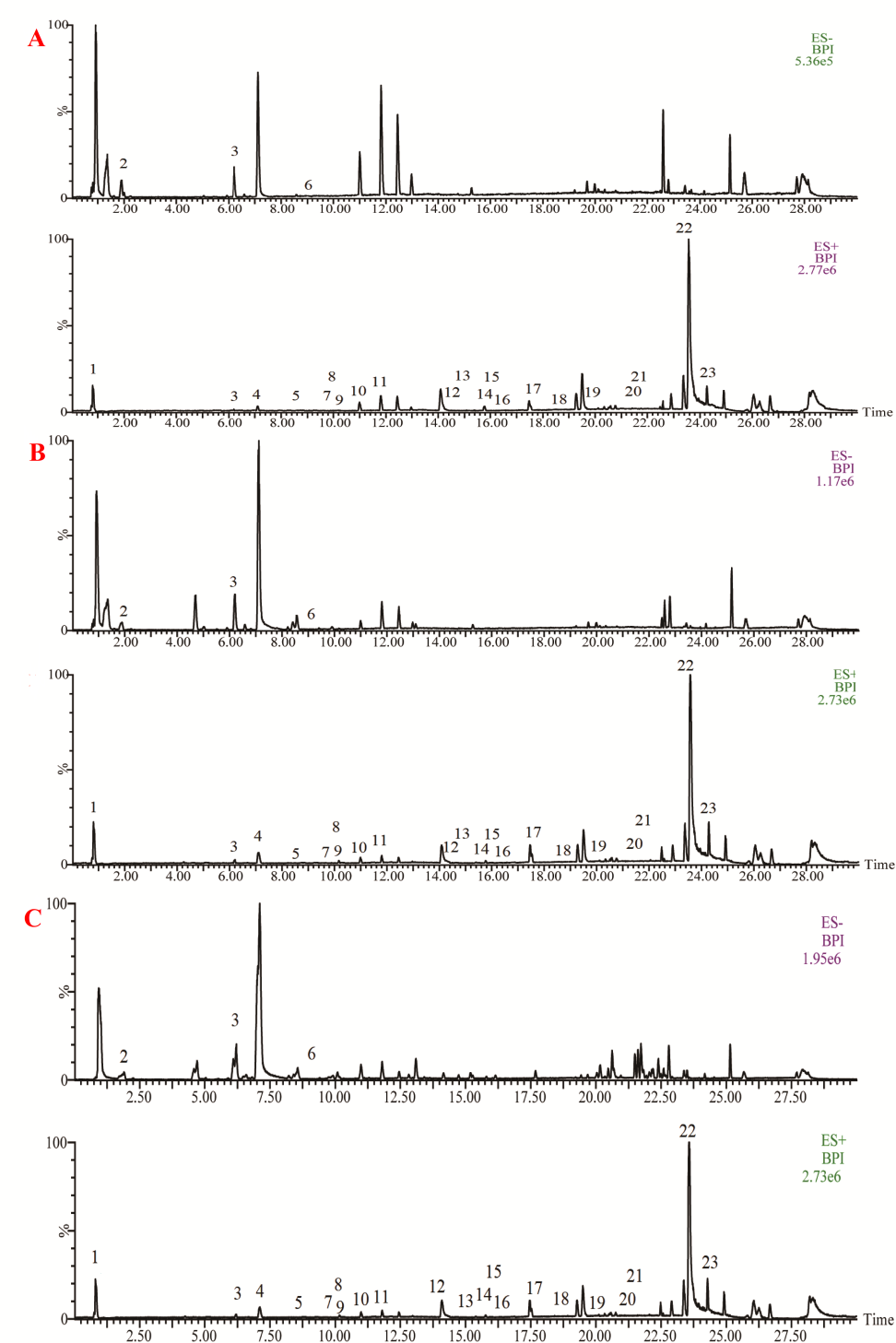
**

**Figure S2. MS fingerprint of three extracts.** (A) WE extract (B) HME extract (C)TME extract. (Retention time of some chemical substances is as the following: 1. Adenosine: 0.927min, 2. Gallic acid: 1.901min, 3. Albiflorin: 6.198min, 4. Paeoniflorin: 7.180min, 5. Galloylpaeoniflorin: 8.552min, 6. Ferulic acid: 9.064min, 7. Senkyunolide I: 9.786min, 8. Mudanpioside I: 10.092min, 9. Lactiflorin: 10.164min, 10. Cyclohexaisoleucine: 10.985min, 11. Cycloheptassic-isoleucine: 11.778min, 12. Senkyunolide A: 14.641min, 13. 3-Butylphthalide: 14.922min, 14. Z-Ligustilide: 15.740min, 15. (E)-Ligustilide: 16.178min, 16. Cnidilide A: 16.377min, 17. [Alisol C-23-acetate](https://www.chemsrc.com/en/cas/26575-93-9_69307.html): 17.682min, 18. Atractylenolide I: 18.763min, 19. Isomaltopaeoniflorin: 20.040min, 20. [Alisol C](https://www.chemsrc.com/en/cas/30489-27-1_1498317.html): 21.484min, 21. Alisol-B-23-acetate: 21.605min, 22. Riligustilide: 23.565min, 23.Tokinolide B: 24.287min)


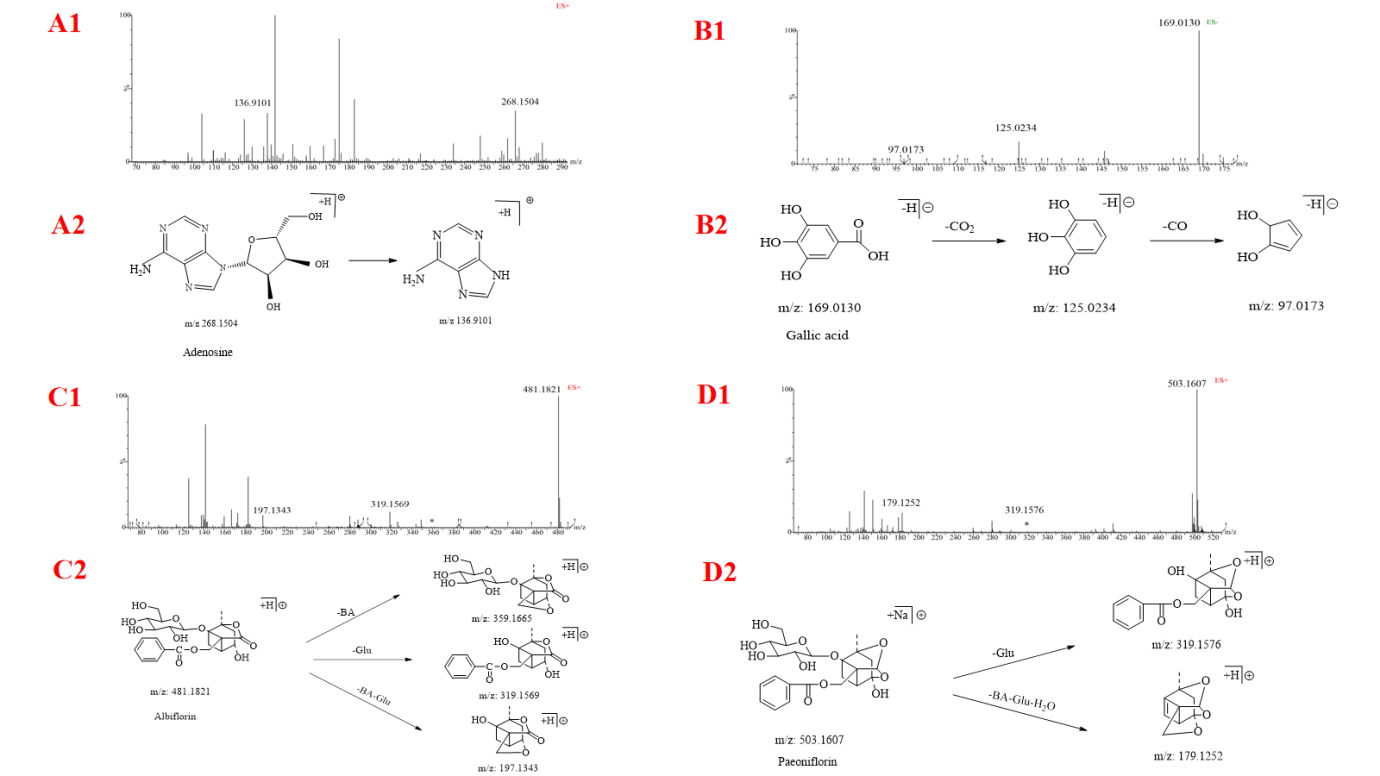


**Figure S3.** MS/MS mass spectra（1） and Mass spectrometric cleavage pathway （2）of each compounds**. (**A**)** Adenosine (B) Gallic acid (C) Albiflorin (D) Paeoniflorin.


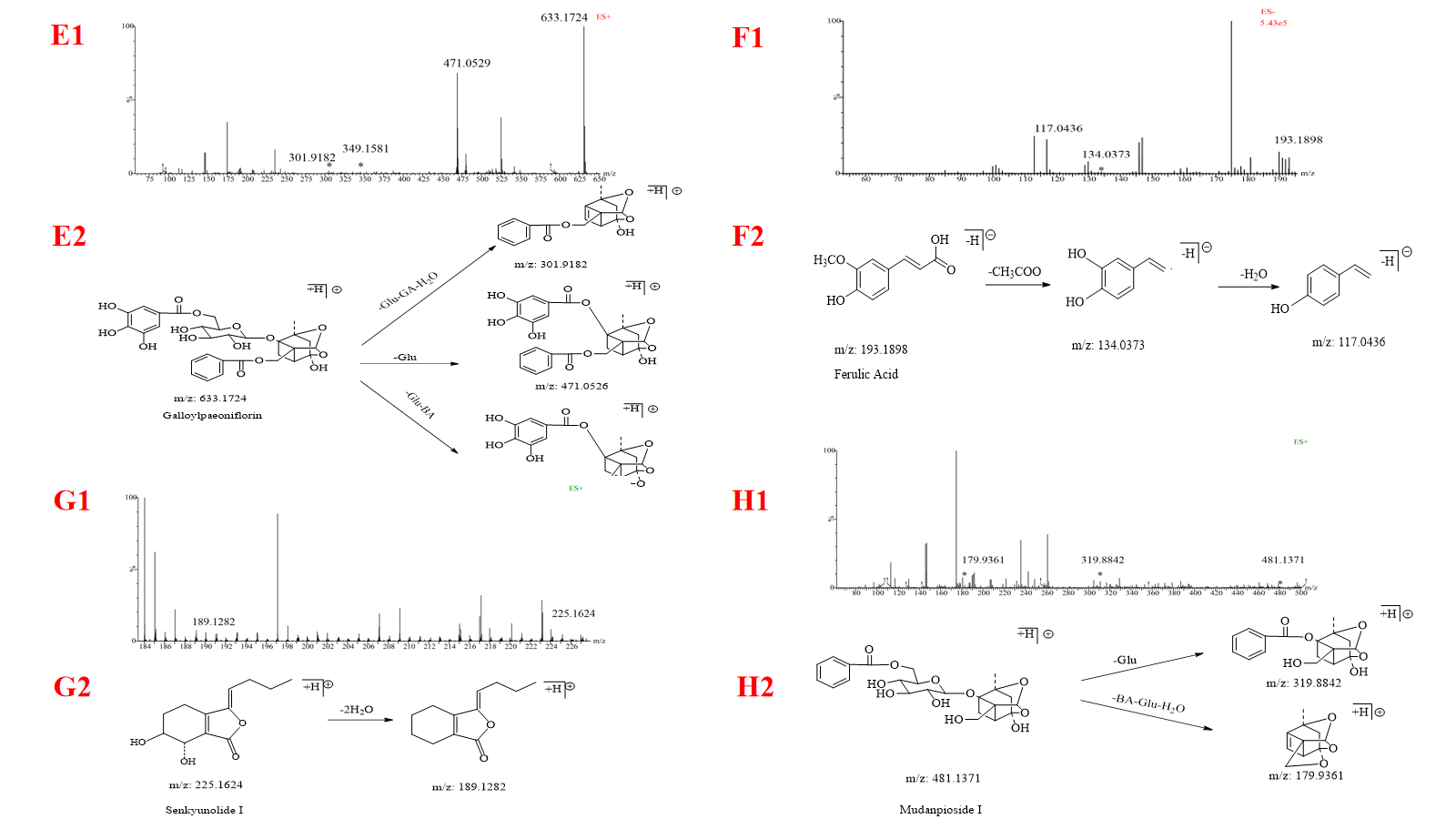


**Figure S4.** MS/MS mass spectra（1） and Mass spectrometric cleavage pathway （2）of each compounds. (E) Galloylpaeoniflorin (F) Ferulic acid (G) Senkyunolide I (H) Mudanpioside I

**
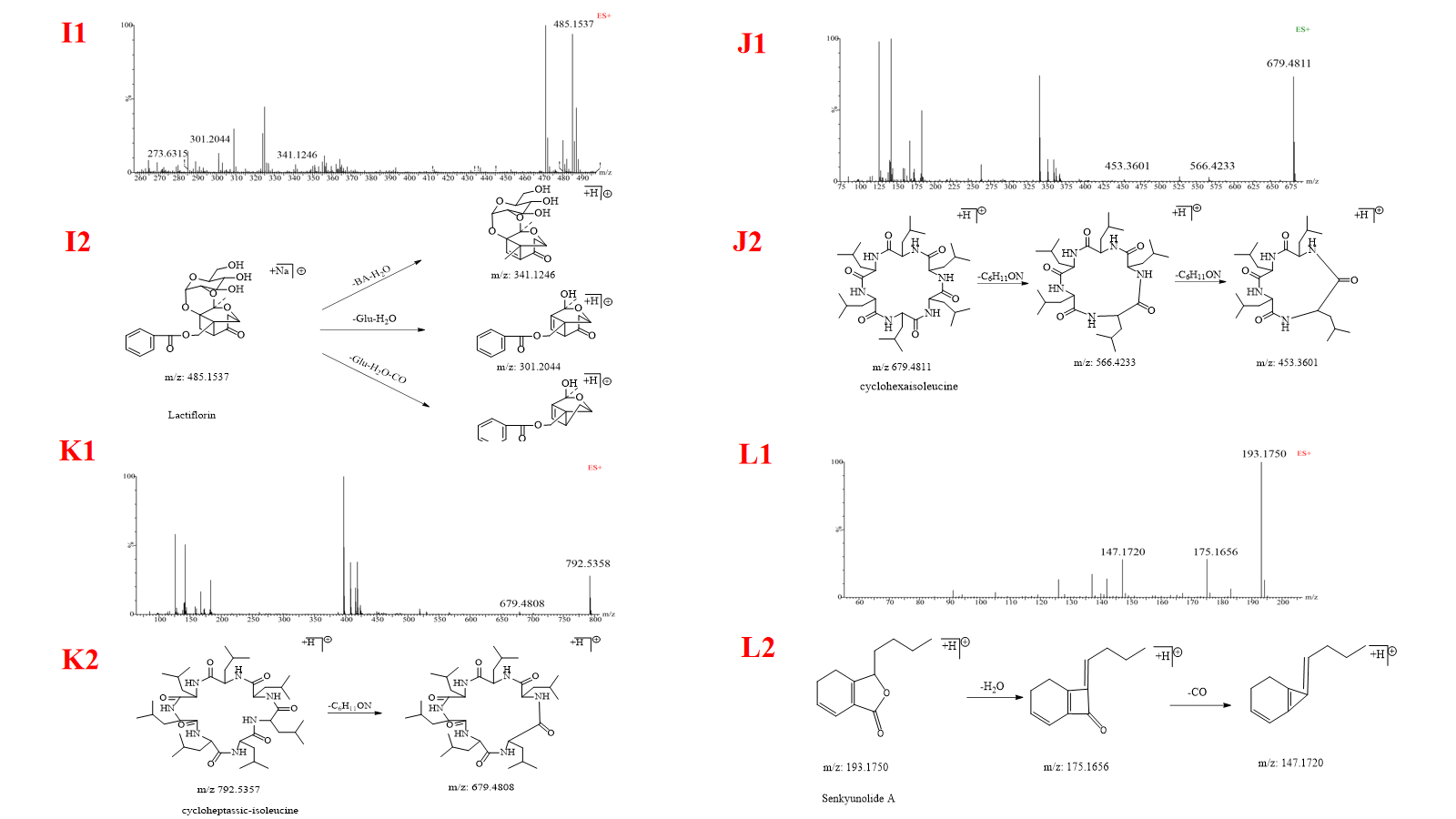
**

**Figure S5.** MS/MS mass spectra（1） and Mass spectrometric cleavage pathway （2）of each compounds. (I)Lactiflorin (J)Cyclohexaisoleucine (K)Cycloheptassic-isoleucine (L) Senkyunolide A

**
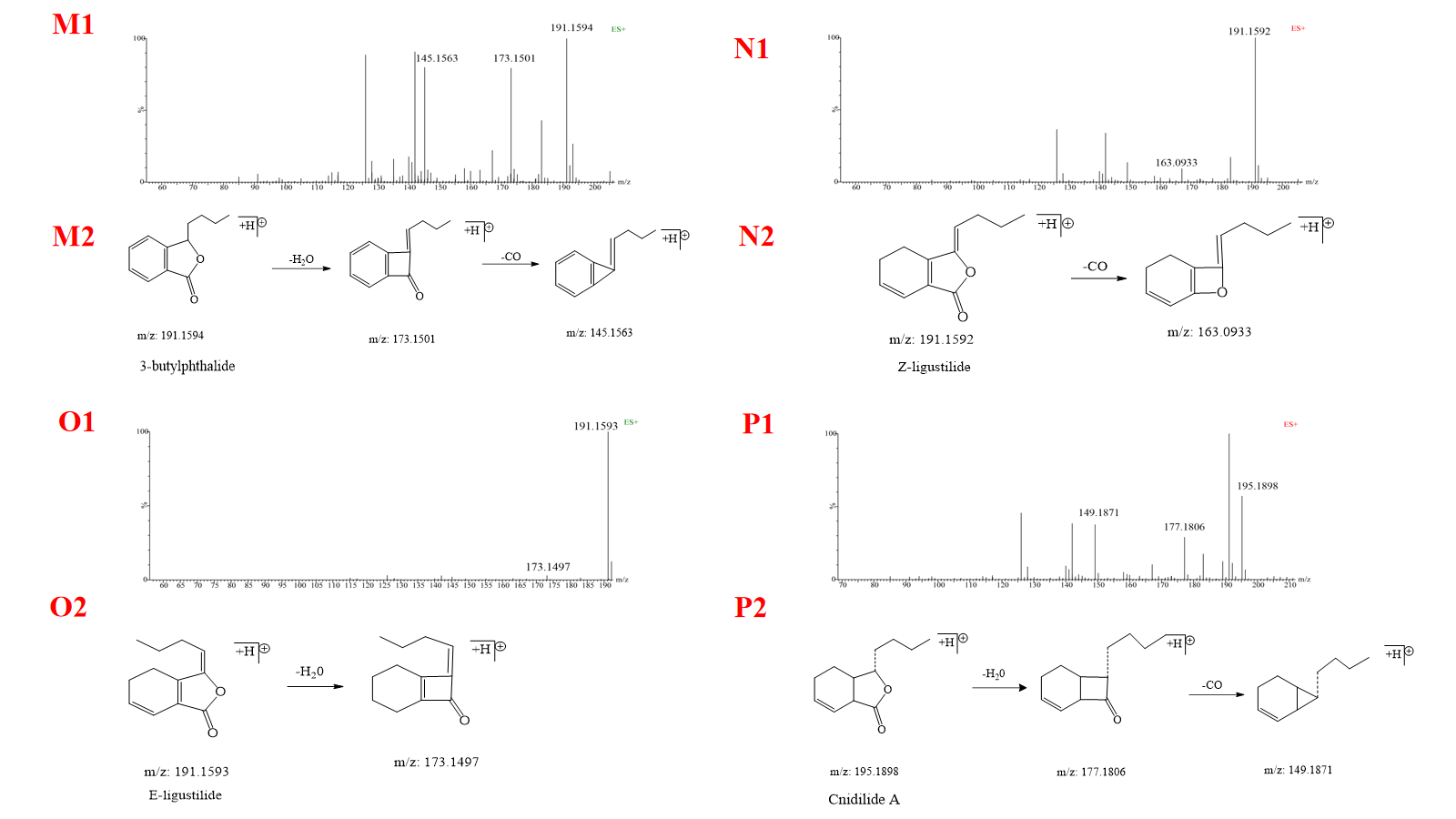
**

**Figure S6.** MS/MS mass spectra（1） and Mass spectrometric cleavage pathway （2）of each compounds. (M)3-Butylphthalide (N) Z-Ligustilide (O) (E)-Ligustilide (P)Cnidilide A

**
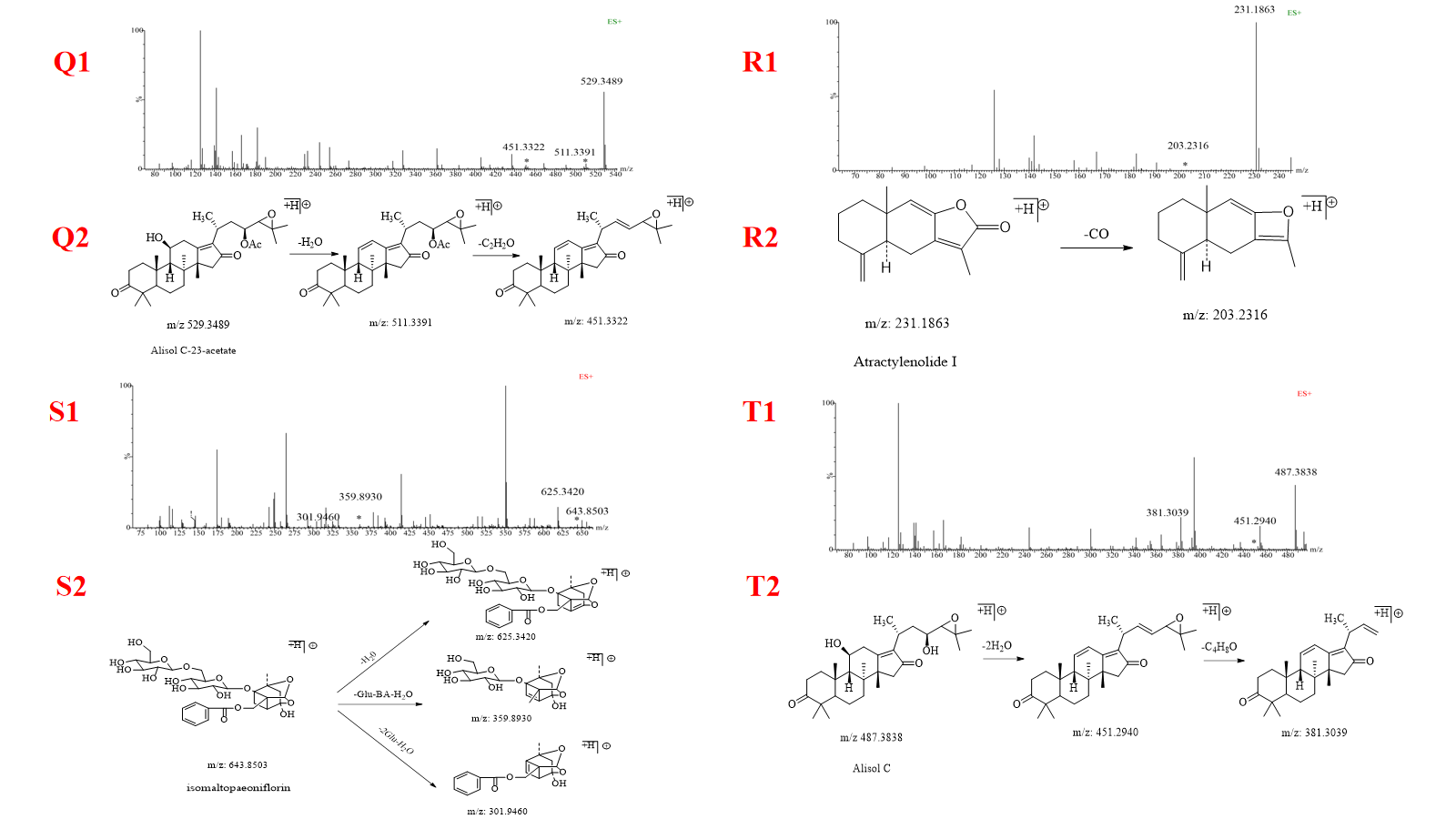
**

**Figure S7.** MS/MS mass spectra（1） and Mass spectrometric cleavage pathway （2）of each compounds. (Q)[Alisol C-23-acetate](https://www.chemsrc.com/en/cas/26575-93-9_69307.html) (R)Atractylenolide I (S)Isomaltopaeoniflorin (T)[Alisol C](https://www.chemsrc.com/en/cas/30489-27-1_1498317.html)

**
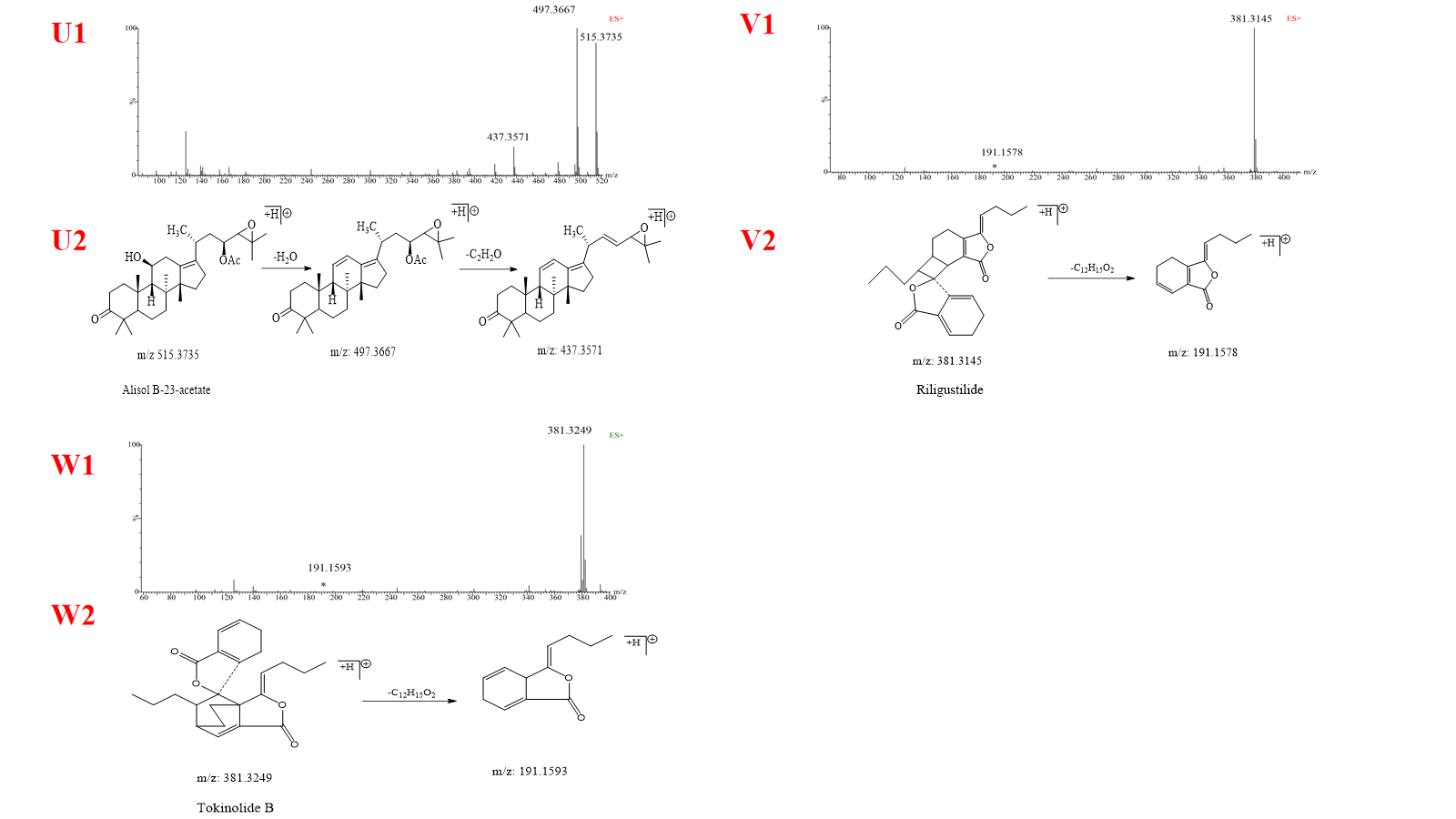
**

**Figure S8.** MS/MS mass spectra（1） and Mass spectrometric cleavage pathway （2）of each compounds. (U)Alisol-B-23-acetate (V) Riligustilide (W)Tokinolide B

**
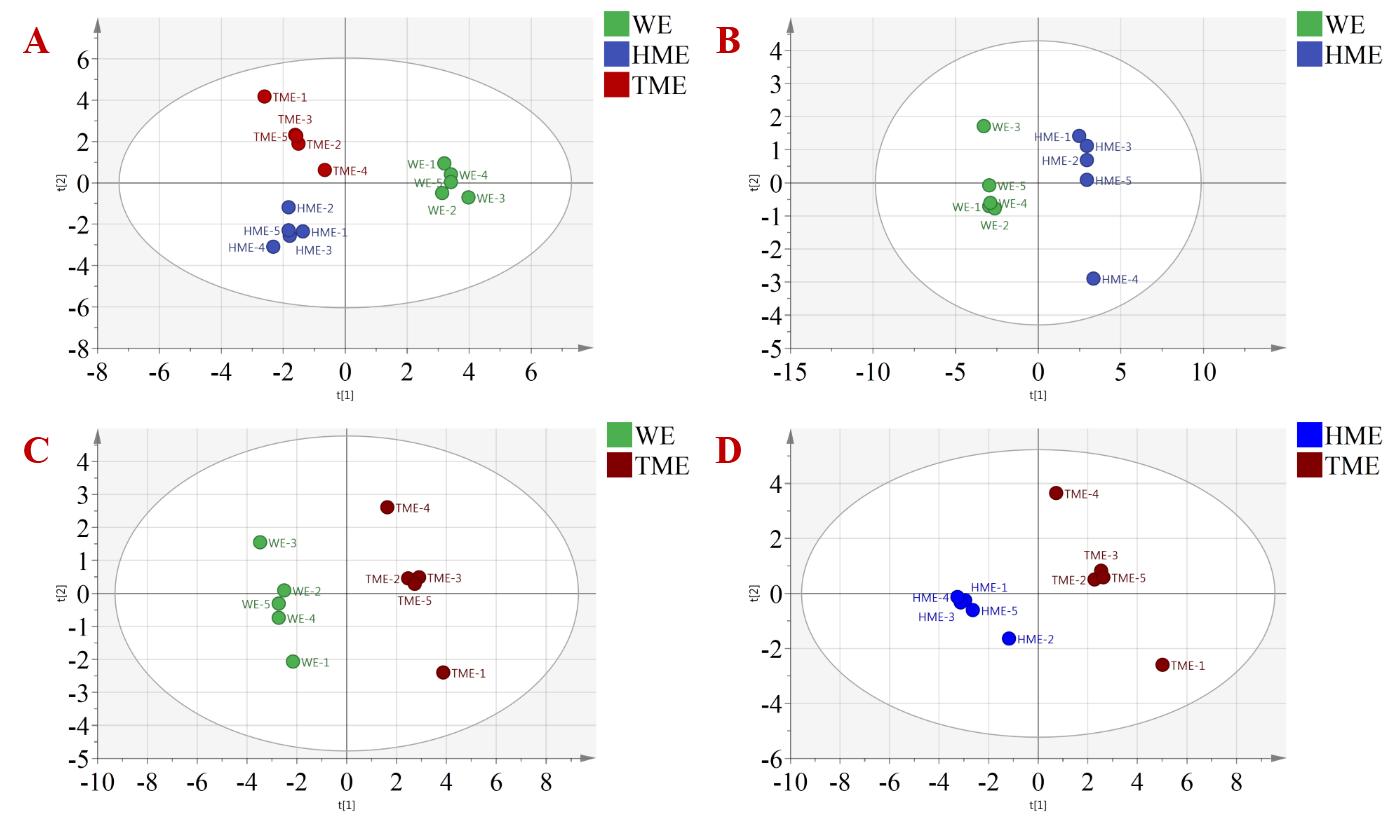
**

**Figure S9. PLS-DA analysis of relative concentration of chemical compounds between different extracts.** (A) PLS-DA analysis among three groups. (B) PLS-DA analysis between WE and HME group. (C) PLS-DA analysis between WE and TME group. (D) PLS-DA analysis between HME and TME group.





**Figure S10. Histogram of relative concentration about common differential chemical substances between WE group and HME/TME group** (**p* < 0.05, ***p* < 0.01, ****p* < 0.001).

**Table S1 Gut microbiota changes related to DSS’s mechanism（Mean±SD）**

| Genus | control | model | HME |
| --- | --- | --- | --- |
| Akkermansia | 0.0084±0.01801 | 0.0023±0.00166 | 0.206±0.17102 |
| Lachnospiraceae_  NK4A136_group | 0.0291±0.00947 | 0.0328±0.05107 | 0.0183±0.01182 |
| Lactobacillus | 0.5772±0.0599 | 0.3388±0.06063 | 0.2023±0.05163 |
| Prevotella_9 | 0.0052±0.00152 | 0.015±0.02084 | 0.0432±0.06747 |
| Turicibacter | 0.0001±0.00005 | 0.0489±0.03639 | 0.1052±0.1038 |
| uncultured_bacterium_f_  Erysipelotrichaceae | 0.0004±0.00024 | 0.0209±0.0265 | 0.0018±0.00206 |

**Table S2 Transcriptomic changes in liver related to DSS’s mechanism**

| Gene ID | Base Mean | Absolute FC≥0.5 | *p＜0.05* | *Q ≤0.01* | Diff | comparision |
| --- | --- | --- | --- | --- | --- | --- |
| ENSRNOG00000002826  (HSD17β7) | 654.9 | -0.91 | 5.3E-10 | 8.2E-08 | Down | control VS model |
|  | 599.0 | 0.69 | 0.00094 | 0.039 | Up | model VS HME |
| ENSRNOG00000002212  (HSD17β13) | 4970.5 | 0.61 | 0.00033 | 0.0085 | Up | control VS model |
|  | 5017.1 | -0.59 | 0.00037 | 0.02 | Down | model VS HME |
| ENSRNOG00000010165  (TNF-α) | 1025.0 | 0.69 | 6.2E-05 | 0.0022 | Up | control VS model |
|  | 1036.9 | -0.67 | 8.3E-06 | 0.00081 | Down | model VS HME |
| ENSRNOG00000060745  (NLRP12) | 927.2 | 0.54 | 0.00075 | 0.016 | Up | control VS model |
|  | 1260.7 | 0.34 | 0.051 | 0.46 | noDEG | model VS HME |
| ENSRNOG00000016456 (IL-33) | 1489.3 | -1.48 | 2.3E-31 | 3.2E-28 | Down | control VS model |
|  | 1017.5 | 0.67 | 0.00013 | 0.0081 | Up | model VS HME |

**Table S3 Hepatic metabolites changes related to DSS’s mechanism（Mean±SD）**

| Metabolites | control | model | HME |
| --- | --- | --- | --- |
| Isoleucine* | 4.23±1.24 | 4.73±1.75 | 6.64±2 |
| Leucine* | 6.81±2.24 | 7.9±3.01 | 12.49±4.54 |
| Valine* | 1.77±0.59 | 1.8±0.53 | 2.58±0.92 |
| lactate | 66.94±20.16 | 85.37±27.32 | 48.98±16.19 |
| alanine | 25.36±10.75 | 39.87±15.3 | 29.66±13.06 |
| acetate | 1.93±0.49 | 2.11±0.72 | 3.04±1.08 |
| glycine | 70.23±23.75 | 101.87±37.75 | 86.13±25.35 |
| gluctose | 44.32±16.03 | 76±26.45 | 48.31±13.48 |
| fumarate | 0.05±0.04 | 0±0.01 | 0.04±0.03 |
| formate | 0.26±0.12 | 0.13±0.14 | 0.21±0.12 |

*note: branched amino acids (BCAAs) are composed of valine, leucine and isoleucine.
